# Accurate Morphology Preserving Segmentation of Overlapping Cells based on Active Contours

**DOI:** 10.1038/srep32412

**Published:** 2016-08-26

**Authors:** Csaba Molnar, Ian H. Jermyn, Zoltan Kato, Vesa Rahkama, Päivi Östling, Piia Mikkonen, Vilja Pietiäinen, Peter Horvath

**Affiliations:** 1 Synthetic and System Biology Unit, Biological Research Centre of the Hungarian Academy of Sciences, Szeged, Hungary; 2 Department of Mathematical Sciences, Durham University, Durham, UK; 3 Department of Mathematics and Informatics, J. Selye University, Komarno, Slovakia; 4 Institute for Molecular Medicine Finland, University of Helsinki, Helsinki, Finland

## Abstract

The identification of fluorescently stained cell nuclei is the basis of cell detection, segmentation, and feature extraction in high content microscopy experiments. The nuclear morphology of single cells is also one of the essential indicators of phenotypic variation. However, the cells used in experiments can lose their contact inhibition, and can therefore pile up on top of each other, making the detection of single cells extremely challenging using current segmentation methods. The model we present here can detect cell nuclei and their morphology even in high-confluency cell cultures with many overlapping cell nuclei. We combine the “gas of near circles” active contour model, which favors circular shapes but allows slight variations around them, with a new data model. This captures a common property of many microscopic imaging techniques: the intensities from superposed nuclei are additive, so that two overlapping nuclei, for example, have a total intensity that is approximately double the intensity of a single nucleus. We demonstrate the power of our method on microscopic images of cells, comparing the results with those obtained from a widely used approach, and with manual image segmentations by experts.

High content analysis of microscopic images is a very active field in computational cell biology^1^,^2^,^3^,^4^,^5^. While many methods have been developed, the analysis of cell cultures and tissue sections at the single-cell level remains a major challenge. As knowledge of cell-level heterogeneity plays a crucial role in improving the understanding and treatment of human diseases such as cancer, there is an urgent need for methods capable of precisely analyzing images of complex cellular phenotypes at single cell-level.

Accurate cell segmentation is the basis of all such analysis, for example the identification of cellular compartments, or feature extraction based on cell morphology, intensity, or texture (Fig. 1). As a result, a great variety of single cell detection algorithms have been proposed. Most simple segmentation methods use local or global thresholding, usually based on the histogram of image intensities, and have therefore the smallest computational requirements^6^,^7^,^8^,^9^. Other methods utilize inherent properties of the image intensity values, such as texture, to detect cells with characteristic patterns^10^. Supervised^11^,^12^,^13^ and unsupervised^14^,^15^ machine learning methods have proven their practical usefulness in single-cell detection applications: they largely outperform classical segmentation techniques by combining multi-parametric image-derived information and non-trivial decision surfaces. However, these single-cell methods often fail to detect multiple cells in complex spatial arrangements. A possible way to overcome this limitation is to incorporate prior shape information about the objects of interest into the segmentation algorithm. A common approach is to fit rigid predefined shapes (i.e. templates) to the image and identify the best matches^16^,^17^,^18^,^19^,^20^. These methods can, to a certain extent, handle overlapping objects, but they are unable to capture small shape variations such as slightly elongations, which may encode essential phenotypic information. An alternative approach, “active contours”, have proven their popularity and usefulness in medical image analysis^21^, but the simplest models do not work well on the difficult problems addressed here. However, it is possible to extend simple active contour models, and incorporate different complexities of prior information about the region of interest^22^,^23^. In particular, the “gas of near circles” model was designed to detect multiple near-circular objects^24^.

In recent years, there has been a growing interest in both academia and industry in developing more complex three dimensional cell culture models. These can better capture the complexity of the tissue, and have the potential to provide more biologically relevant information than two-dimensional models^25^,^26^,^27^. The conventional epifluorescence high-content microscope visualization is often used for shRNA, CRISPR-Cas9 and drug-screening of such 3D cultures, but in these cases it results in images containing many overlapping cells/nuclei. In addition, aggressively growing tumor cells, which have lost contact inhibition; co-cultures of different cell types in 2D; and fluorescently stained tissue samples provide similar challenges. The segmentation methods cited above are not capable of precisely detecting cell nuclei in these cases. Here we present a novel segmentation method, extending the “multi-layer gas of near-circles” (MLGOC) model of Molnar and colleagues^28^, that can be successfully applied to the counting of overlapping nuclei and to the determination of exact nuclear morphologies from fluorescence microscopy images. Our method uses an important property of most conventional wide-field fluorescence microscopy images: the intensity measured by the microscope at a location is proportional to the density of fluorescent particles, and therefore we assume that using low numerical aperture objectives, the intensity contribution of cells growing on top of each other is approximately the sum of the individual cell contributions. We present a new data term that captures this property and incorporate it into the MLGOC framework. The resulting model is capable of segmenting overlapping nuclei while preserving their morphologies, thus providing a unique platform for high-throughput analysis.

We validate the method on synthetic and manually labeled sets of images of a prostate cancer cell line with fluorescently stained nuclei, comparing the results with those obtained from widely-used methods for the segmentation and analysis of single cells.

## Methods

### Cell preparation and imaging

The PC346C prostate cancer cell line used for the validation experiments was obtained from the Erasmus Medical Centre in Rotterdam^29^,^30^. 2000 cells in 25 *μ*l of complete medium were delivered to the wells of 384-well plate with the Multidrop Combi Reagent Dispenser (Thermo Fisher Scientific Oy, Finland) using a standard cassette (Thermo Fisher Scientific Oy). After 72 hours, cells were fixed with 4% paraformaldehyde, and stained for 10 min at room temperature with Hoechst33324 (Life Technologies; stock 20 mg/ml, diluted to phosphate-buffered saline, 1:20 000) to detect the cell nuclei. All washing steps were performed with the EL406 Combination Washer Dispenser (Biotek, Germany). The images of fluorescently stained cells were captured with the automated epifluorescence microscope ScanR (Olympus, Germany) with a 150 W Mercury-Xenon mixed gas arc burner, a 20x/0.45 N.A. long working distance objective (Olympus), with 5 ms exposure time (UV channel) without binning, and a 12-bit (1344 × 1024, horizontal × vertical pixels) digital monochrome interline transfer CCD camera C8484-03G01 (Hamamatsu), cooled with the Peltier element. The pixel size at 20x objective is 0.323 *μ*m/pixel.

### Image formation model for overlapping cell nuclei

The image formation model we use is *I*_observed_ = *I*_background_ + *I*_original_, where it is assumed that illumination problems have already been corrected^31^. *I*_background_ is a nearly flat non-zero surface with noise, so called “dark noise”. As described in the Introduction, using low numerical aperture objectives we assume that measured intensity is proportional to the density of fluorescent particles. This means, for example, that the intensity contribution of two cells on top of each other is approximately double that of a single cell. Let *μ*_−_ and 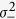 be the mean and variance of the background intensity, and *μ*_+_ and 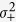 be the mean and variance of the measured intensity of a single cell. Let 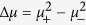, and 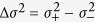. Then, according to the model, the mean of the intensity of multiple cells is given by *μ*_−_ + *n*Δ*μ*, and its variance by 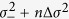. The parameters *μ*_−_, 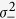, Δ*μ*, and Δ*σ*^2^ are estimated from the corrected images using maximum likelihood estimation.

### “Multi-layer gas of near circles” model

Active contours are popular image segmentation models^21^ that describe regions by their boundaries. An energy function of this boundary is defined that encodes information about boundary shape (the “geometric term”), to which is then added a “data term” in order to perform segmentation. We describe our geometric term first.

In the simplest geometric term, the only interaction is between neighboring points. This allows the model to describe restrictions on boundary length and object area:


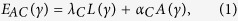


where *γ* is the representation of the region boundary, and *L* and *A* are boundary length and object area, with weight parameters *λ*_*C*_ and *α*_*C*_, respectively. This ‘classical’ active contour model has low energy when the object’s area is small and its boundary is smooth.

In contrast to this simple model, “higher-order active contour” models use multiple integrals over the boundary in the geometric term. These express long-range interactions between points on the boundary, and therefore can incorporate more specific information about object shape. One of the simplest of these higher-order functionals is^32^:


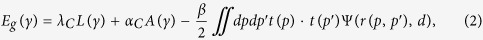


where *t* is the tangent vector to the contour; *p* and *p*′ are contour parameters; and *r*(*p*, *p*′) is the distance between the points *γ*(*p*) and *γ*(*p*′). The interaction function Ψ is a monotonically decreasing function that controls the nature of the long-range interaction; it has a parameter *d* that controls the interaction range. A special parameterization of this higher-order model, the “gas of circles” (GOC) model^33^, assigns low energy to configurations consisting of several near-circles with approximately a given radius.

Rather than represent the object boundary directly, as a parameterized curve, it is convenient to use a level set representation^34^ known as a “phase field”. The phase field formulation of a model can be used as an equivalent alternative to an active contour formulation^35^. It possesses many advantages, including easy handling of complex topologies and low computational cost. A phase field represents a subset 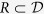 by a function 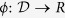 on the image domain 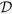, and a threshold 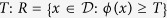. The phase field geometric term equivalent to *E*_*g*_ can be written in the form^36^:


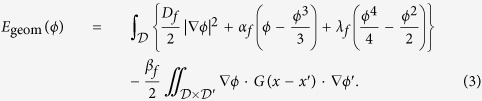


The above model, whether in its active contour or phase field formulation, has two main limitations. The first one comes from the representation: like most segmentation methods, it cannot represent overlapping object instances. The second arises from the geometric model itself: the non-local energy term, which causes the model to favor near-circular shapes, also results in a repulsive force between neighboring objects separated by a distance comparable to the desired object size. As a result, this method cannot effectively handle objects that are close to each other or overlapping.

An extended version of the GOC approach^28^ uses several independent layers of phase fields to overcome these limitations. The geometric term of the model is simply the sum of the geometric terms of the individual layers, extended by a term that penalizes overlap. However, the overlap penalty was only introduced to solve a problem created by the data term used by Molnar *et al*.^28^, which tried to match each layer of the segmentation separately to the image data, sometimes resulting in the same object being segmented multiple times in different layers; the overlap penalty helped to avoid this. The current model tries to match a combination of the individual segmentation layers to the data, and assumes that the measured intensities are a (noisy) additive function of the number of overlapping objects; as a result, these degenerate segmentations do not occur, and we can set the overlap penalty to zero without any negative effects. Overlapping object instances can now be represented by appearing in different layers, while repulsion between objects in different layers is eliminated, even if close or overlapping, since the geometric term contains no inter-layer interactions.

### New data model for fluorescent microscopy

In this section, we introduce a new data model that is adapted to the image formation process in fluorescence imaging. The new model is constructed using the assumption that overlapping cells contribute additively to the image intensity, so that multiple cells on top of each other produce an intensity contribution that is a multiple of that of a single cell. (An early version of this work was presented recently^37^).

**Figure 2:**
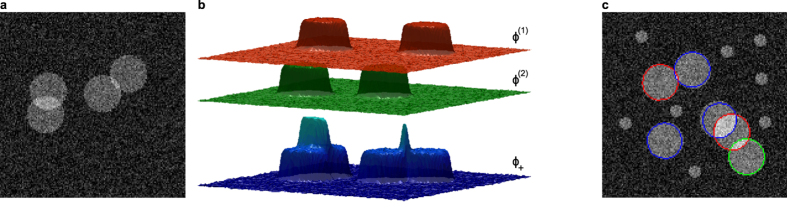
Illustration of the proposed data model and behavior of the geometric model. (**a**) Noisy synthetic image. (**b**) Phase field representation of the cell configuration in image (**a**), showing the two layers, and the combined *ϕ*_+_ function that “counts” cells. (**c**) Size selectivity of the “gas of circles” model: using proper settings of the prior and data parameters, it is possible to achieve size-selective segmentation. No initial object seeds were used.

Let 
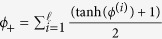
 (Fig. 2), where *ϕ*^(*i*)^ is the phase field in the *i*^th^ layer; this quantity “counts” the number of cells at each point. Let *γ*_*d*_ be the (positive) weight of the data term; and let *I* be the intensity of the input image. Using the image formation model described earlier, and a Gaussian model for the image noise, the new data term becomes:


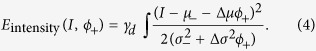


Since a phase field takes the values −1 and 1 in its two phases (background and foreground), with a smooth transition between them, the integrand in Eq. (4), which is the energy density, takes a low value when *ϕ*_+_ = 0 over regions with background intensity; *ϕ*_+_ = 1 over regions of single-cell intensity; and generally, when *ϕ*_+_ = *n* over regions with *n* cell intensity.

We use gradient descent in order to minimize the overall energy 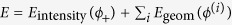 and find the optimal phase field configuration, and hence segmentation. The functional derivative of *E*_geom_ is given in Molnar *et al*.^28^. The functional derivative of *E*_intensity_ is:


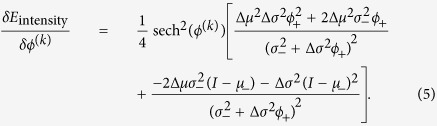


### Dependence on initialization

Gradient descent methods search for a local optimum of the energy function. The initialization is therefore crucial: a good initialization can greatly increase the accuracy of the resulting segmentation. We use two initialization methods:

- “Neutral initialization” means that the initial phase field is a realization of Gaussian white noise with mean *λ*_*f*_/*α*_*f*_ and a small variance.
- “Seeded initialization” means that we have estimates of the “centers” of the objects, the “seeds”. These could be computed by any available method; they are currently given manually. The initial phase field is defined to be 1 at all points within half the preferred radius of a seed, and zero elsewhere. The seeds are distributed among the layers so as to minimize overlaps.

### Evaluation method and metrics

To evaluate the algorithm and to compare to other methods, three metrics were used. The first two metrics are the precision and recall of object detection. Precision (or positive prediction value) is the ratio of true positives (TP) to the number of detected objects (Precision = TP/(TP + FP)). Recall (or sensitivity) shows what proportion of the objects of interest is found (Recall = TP/(TP + FN)). Values closer to 1 imply better detection. Values are computed as follows. First, a matching is made between the set of ground truth objects and the set of segmented objects. Objects with no overlaps with any other object are deemed not matched. A weight is then assigned to each pair of remaining segmented and ground truth objects, equal to the reciprocal of the area of overlap. The total weight of a matching is defined as the sum of the weights of the matched pairs. This is an instance of the assignment problem; we solve it with the Hungarian algorithm. TP is then the number of segmented objects that have a matching ground truth object, while FP is the number of segmented objects that have no matching ground truth object. FN is the number of ground truth objects that have no matching segmented object. Note that these metrics do not measure morphological accuracy. The third metric measures the morphological accuracy of the segmentations of the individual, correctly-detected objects. For each matched pair of segmented (A) and ground truth (B) objects, we compute the Jaccard index, the ratio of the area of their intersection to the area of their union: 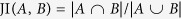. The final measure is then the average of the Jaccard indices of the matched pairs.

### Annotated images of a prostate cancer cell line

To test our model, twenty fluorescence microscopy images of different degrees of complexity, containing ~2000 cells in total, were chosen for quantitative evaluation. For evaluation purposes, ground truth segmentations were generated by manually annotating the images. The annotations were made by two experts using ImageJ ROI Manager with Freehand selection. The image set consists of 20 images: 18 are different images with varying degrees of complexity; the other two images are 180°-rotated copies of two randomly chosen images from the initial 18.

## Results

### Synthetic data sets

In order to measure the robustness of the proposed method, we generated data sets of synthetic images with different values for the noise variance, the extent of overlap, and varying object ellipticity and size, to create variability similar to that seen in real world observations.

#### Object size selectivity

To analyze the ability of our model to select objects of the correct size, a set of images was generated containing circles of two radii: 5 and 15. The expected radius of the GOC model was set to the radius of the larger objects. Figure 2c illustrates the results obtained using the model with the neutral initialization. The proposed model was able to select circles with the desired radius and correctly separate overlapping objects, while at the same time eliminating the circles with the smaller radius.

#### Dependence on initialization

**Figure 3:**
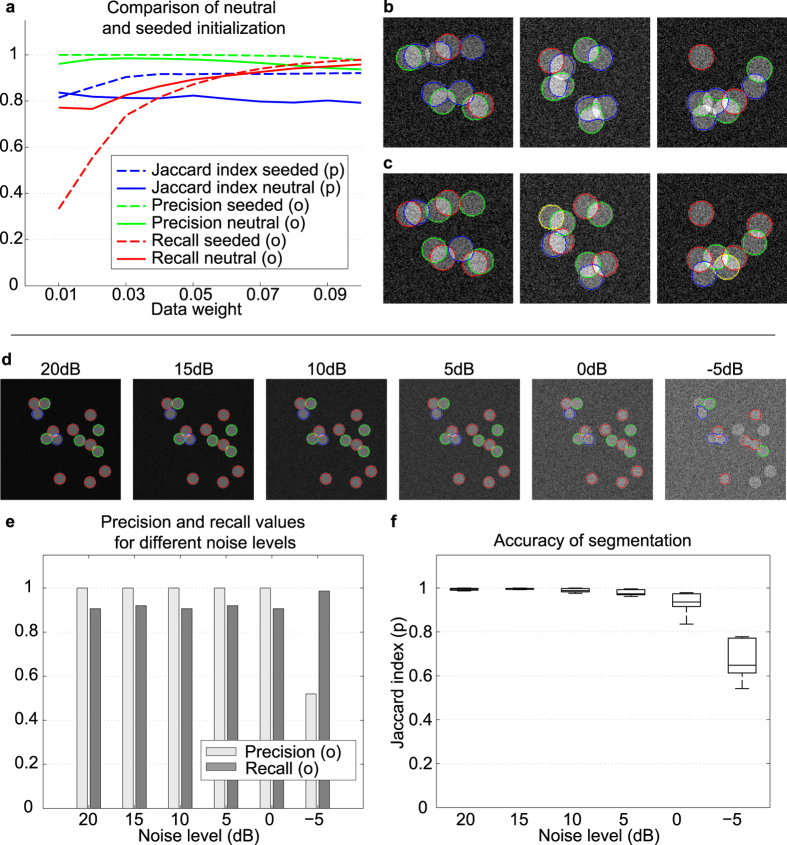
Synthetic results with different initialization methods and the robustness of the model to noise. (**a**) Evaluation of segmentations with different initialization methods. “Neutral” means the initial *ϕ* was set to a Gaussian white noise with mean *λ*_*f*_/*α*_*f*_ and small variance (no initial objects are needed). “Seeded” means that small circles inside the objects were used to initialize the phase field. (**b**) Segmentation results with neutral initialization using 3 layers. (**c**) Segmentation results with seeded initialization. (**d**) Synthetic image with overlapping circles and increasing levels of signal-to-noise ratio (+20 dB to −5 dB). (**e**,**f**) Evaluation of the results of the proposed model. The segmentation results were evaluated on 50 test images per noise level. Note that different data term weights *γ*_*d*_ were used for different noise levels. (**e**) Average precision and recall values of segmentation results for different signal-to-noise ratios. (**f**) Box and whisker plot of segmentation accuracy for different levels of signal-to-noise ratio. The bottom and top edges of the box indicate the first and third quartiles; the line inside the box indicates the median; the whiskers (lines protruding from the box) indicate the smallest and largest data points whose distance from the box is not greater than 1.5 times the interquartile range.

To compare “Neutral” and “Seeded” initialization methods, 60 synthetic images (of size 400 × 400 pixels) containing {30, 35, 40} circles of radius 15, and 140 synthetic images (of size 150 × 150) containing 4–10 circles of radius 15 were generated (with 10 dB signal-to-noise ratio level). Background and foreground intensities were chosen from sets {30, 40, 50} and {90, 100, 110} respectively for 8-bit grayscale images. The set of synthetic images thus contains 6300 + 6860 = 13160 perfect circles with different degrees of overlap (0–100%). Figure 3 shows that the manual, seeded initialization makes the model 4–5% more effective at pixel level segmentation accuracy, without loss of object detection accuracy: with an appropriate choice of data weight, the manual initialization performs better than the neutral initialization with respect to all measures.

#### Noise sensitivity

It is common in fluorescence microscopy for images to have low contrast and low signal-to-noise ratio (SNR) because of weak fluorescent staining or microscope properties. In order to show the robustness of the proposed method to noise, 50 synthetic images containing 15 circles with up to 20% overlap were generated. Images were distorted with levels of Gaussian white noise, resulting in SNRs of 20 dB, 15 dB, 10 dB, 5 dB, 0 dB, and −5 dB. Note that 0 dB means that signal and noise have equal power, while −5 dB means that the noise power is roughly three times the signal power. The proposed method was able to segment overlapping circles up to 0 dB with minimal error, with the first errors appearing at SNR = −5 dB (Fig. 3).

#### Separation of overlapping objects

**Figure 4:**
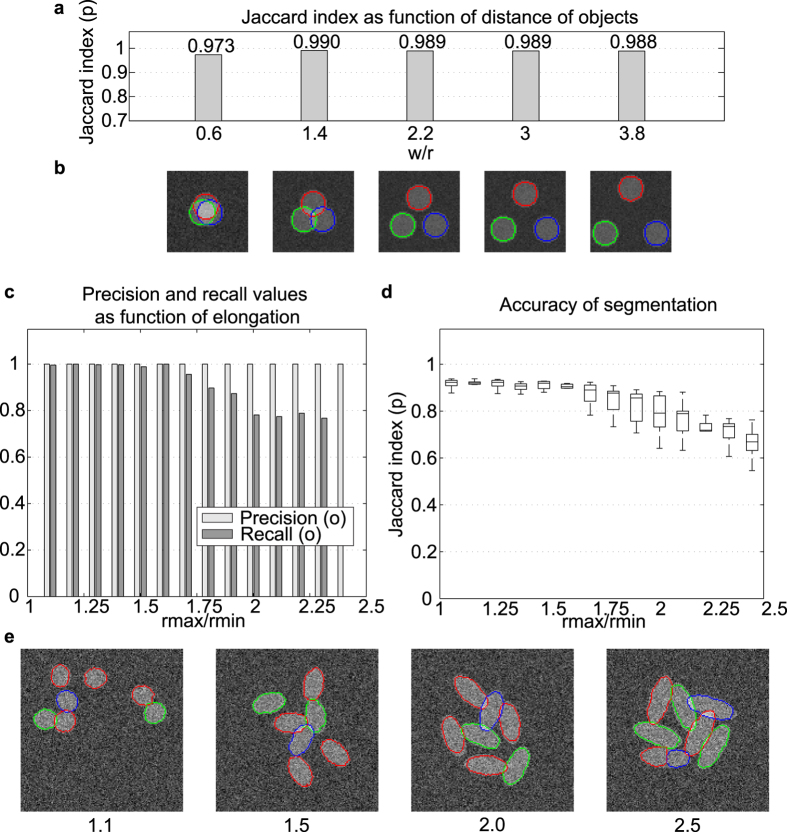
Separation ability of the proposed method and its robustness to elongation of circular objects. (**a**) Segmentation accuracy on synthetic images containing circles with increasing degrees of overlap. Numbers on the x-axis show the *w*/*r* ratio, where *w* is the distance between circles’ centers and *r* is radius of the circles. Values of *w*/*r* < 2 mean that objects are overlapping. (**b**) Examples of segmentations of synthetic images containing circles with different degrees of overlap. (**c**) Average precision and recall values for overlapping ellipses with increasing elongation. The values are computed from 100 test images per value *r*_max_/*r*_min_ = {1.1, 1.2, …, 2.5}. (**d**) Box and whisker plots representing accuracy of segmentation for increasingly elongated ellipses. The bottom and top edges of the box indicate the first and third quartiles; the line inside the box indicates the median; the whiskers (lines protruding from the box) indicate the smallest and largest data points whose distance from the box is not greater than 1.5 times the interquartile range. (**e**) Examples of segmentations of synthetic images containing ellipses of increasing elongation.

In order to test the ability of the model to separate overlapping objects accurately, a series of images was created, with each image containing three overlapping circles of the same radius placed at the corners of an equilateral triangle. The distance between the centers of the circles was varied from 0 to 4 times the radius. Additional noise (SNR = 0 dB) was added to the images. The segmentation results show that the accuracy is independent of the extent of the overlap, and that the method is capable of successfully segmenting circles (Fig. 4).

#### Ellipticity test

The shapes of cell nuclei vary from circles to more elongated elliptical shapes, depending on various biological aspects, such as the origin of the cell and the phase of the cell cycle^38^. For example in our annotated image set, the mode (most frequent value) of the minor and major axes ratio of the nuclei was 1.32, and the variance 0.43. The GOC shape model was originally designed to detect near circular shapes with possible slight perturbations^24^. We tested the ability of the proposed model to capture elongated objects (Fig. 4). Synthetic images were generated containing ellipses with *r*_min_ = 10 and *r*_max_ = {11, 12, …, 25}. The allowed overlap between two ellipsoids was 10%. As expected, the segmentation becomes worse for larger *r*_max_. The proposed method is suited to most conventional cell nuclei types, but we do not recommend its use when the major/minor axis ratio exceeds 1.75.

#### Semi-real tests: SIMCEP

**Figure 5:**
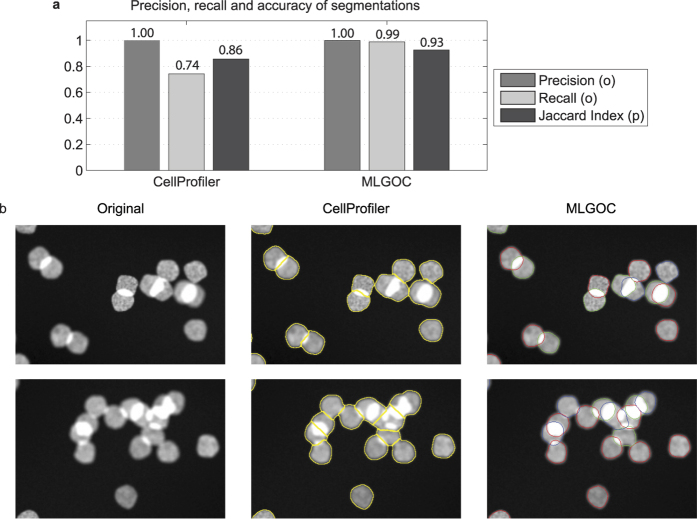
Segmentation results on SIMCEP (framework for generating artificial fluorescent microscopy images) data set. (**a**) Object detection and segmentation accuracy of CellProfiler and the proposed MLGOC method. (**b**) Left column: images containing overlapping artificial nuclei with possible distortion caused by a microscope applied; middle column: segmentations with CellProfiler; right column: segmentations with the proposed method.

To simulate real cell nuclei we used a framework, SIMCEP, designed to generate and to evaluate algorithms for fluorescent microscopy images^39^. 50 images were generated by the framework, each containing 30 nuclei with a 50% maximal allowed overlap. The images were segmented by both CellProfiler and the method proposed here (see Fig. 5). The proposed method outperformed CellProfiler by 5–6% at pixel level accuracy and successfully identified cellular morphology on SIMCEP generated data.

### Real data set

#### Results on annotated images of a prostate cancer cell line

**Figure 6:**
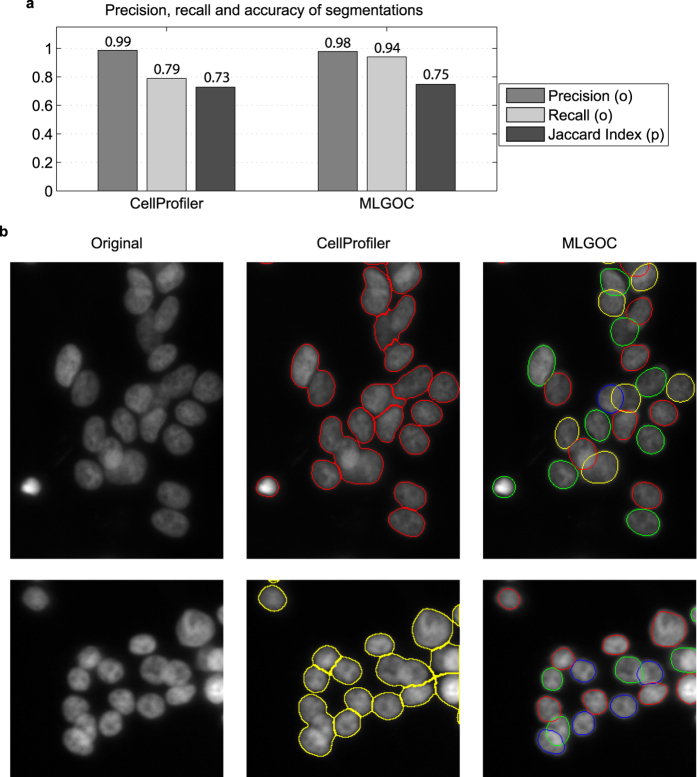
Segmentation results on real fluorescence microscopy images. (**a**) Object detection and segmentation accuracy of CellProfiler and the MLGOC method. (**b**) Left column: original images of prostate cancer cells containing overlapping nuclei; middle column; segmentation results obtained with the CellProfiler software; right column: results with the MLGOC method.

We measured the segmentation accuracy on fluorescence microscopy images using precision/recall and the Jaccard index, and compared the results with those obtained from CellProfiler. In CellProfiler, we used a mixture of a Gaussian global threshold method and an intensity-based splitting of clumped objects with object diameters in the range [25 and 35] pixels. In the MLGOC model, the preferred radius was set empirically to 17 (Fig. 6), the mean radius of the manually annotated nuclei. In comparing the MLGOC method to CellProfiler, we treated each image as different; therefore each of the 20 images has separate ground truth. In measuring self-consistency, we designated the original image as ground truth and the rotated version was compared to it, then vice-versa; the single metrics are the averages of the pairs of measures so obtained. A similar method was used to measure inter-expert agreement. The experts’ self-consistency measurements led to mean precision, recall, and Jaccard index values of 0.98, 0.96 and 0.78 respectively. The values for inter-expert agreement were somewhat weaker: 0.93, 0.93 and 0.75 respectively. As shown in Fig. 6, the proposed method outperforms the segmentation accuracy of CellProfiler, and also achieves a better Jaccard index, the value being as good as that arising from the agreement between different field experts. Separation ability is thus improved without losing detection accuracy.

## Discussion

We have presented a novel method for the segmentation of near-circular objects, such as cell nuclei, from fluorescence microscopy images. Unlike previous methods, the model can handle the most difficult cases involving multiple overlapping objects, while still accurately capturing object morphology. This is of major importance for the analysis of the phenotypic behavior of cell populations. In this paper, the method is adapted to the segmentation, from fluorescence microscopy images, of cell nuclei, but could equally be applied to the segmentation of closely interacting or overlapping intracellular organelles, such as endosomes, lysosomes, or lipid droplets. Our image model, although simple, applies to the conventional imaging systems used for high-content screening and tissue scanning with general 10–40x objectives, and in these cases, and for most conventional cell nuclei morphologies, the proposed method represents a state-of-the-art segmentation tool. The image model also applies to other image modalities with related image formation properties, for example electron microscopy. We anticipate that our method would perform well in these cases also.

In the future, we will investigate new seed initialization techniques, and an adaptive data term that can automatically handle image-to-image intensity variations and illumination differences within the same image.

## Additional Information

**How to cite this article**: Molnar, C. *et al*. Accurate Morphology Preserving Segmentation of Overlapping Cells based on Active Contours. *Sci. Rep.* **6**, 32412; doi: 10.1038/srep32412 (2016).

## Figures and Tables

**Figure 1:**
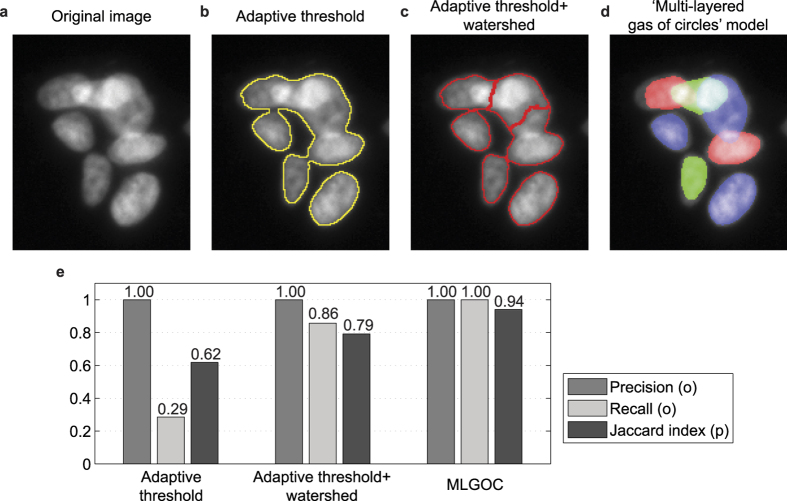
Comparison of different methods on microscopic images containing overlapping cells. Top row from left to right: (**a**) Original image; (**b**) Result (Region of Interest) obtained by adaptive threshold using CellProfiler^7^; (**c**) Results of CellProfiler standard segmentation method; (**d**) Results with the proposed “multi-layer gas of near-circles” method; (**e**) Precision, recall and Jaccard index of segmented objects (‘o’ and ‘p’ indicate that the metrics are computed at the object and pixel level respectively).
